# An international survey assessing the effects of the duration of attack-free period on health-related quality of life for patients with hereditary angioedema

**DOI:** 10.1186/s13023-024-03247-1

**Published:** 2024-06-22

**Authors:** Robbin Itzler, William R. Lumry, John Sears, Julia Braverman, Yinglei Li, Caroline J. Brennan, Gary G. Koch

**Affiliations:** 1 CSL Behring, King of Prussia, PA USA; 2 AARA Research Center, Dallas, TX USA; 3 Research Partnership, Fort Washington, Philadelphia, PA USA; 4 Department of Biostatistics, University of North Carolina at Chapel Hill, Chapel Hill, NC USA

**Keywords:** Angioedema, Hereditary, Humans, Complement C1 inhibitor protein, Quality of life, Prevention and control, Surveys and questionnaires

## Abstract

**Background:**

Hereditary angioedema (HAE) is characterized by unpredictable and often severe cutaneous and mucosal swelling that affects the extremities, face, larynx, gastrointestinal tract, or genitourinary area. Introduction of novel long-term prophylactic treatment options (lanadelumab, berotralstat, and C1-esterase inhibitor SC [human]) into the treatment armamentarium has substantially reduced HAE attacks, allowing patients to be attack free for longer with improvements to their quality of life. Using data drawn from a wide-ranging survey of patients with HAE, we examined the relationship between duration of time attack free and health-related quality of life (HRQoL), exploring the possibility that there is an association between observed improvement in HRQoL and attack-free duration.

**Methods:**

A survey among patients with HAE on long-term prophylaxis (LTP) in six countries (the US, Australia, Canada, UK, Germany, and Japan) assessed the relationship between attack-free duration and mean Angioedema Quality of Life (AE-QoL) scores, quality of life benefits, and rescue medication used. Analysis of covariance (ANCOVA) was used to assess the roles of LTP and attack-free period (< 1 month, 1– < 6 months, ≥ 6 months) on total AE-QoL scores. Results include descriptive *p*-values for strength of association, without control for multiplicity. Descriptive statistics were used to show the relationship between time attack free and quality of life benefits.

**Results:**

Longer durations of time for which participants reported being attack free at the time of the survey correlated with better AE-QoL scores and less use of rescue medication. The mean total AE-QoL scores were 51.8, 33.2, and 19.9 for those who reported having been attack free for < 1 month, 1– < 6 months, and ≥ 6 months, respectively, with higher scores reflecting more impairment. The ANCOVA results showed a strong association between attack-free duration and AE-QoL total score.

**Conclusion:**

This study shows that longer attack-free duration has an influential role for better HRQoL in patients receiving LTP. Prolonging the attack-free period is an important goal of therapy and recent advances in LTP have increased attack-free duration. However, opportunities exist for new treatments to further increase attack-free duration and improve HRQoL for all patients with HAE.

**Supplementary Information:**

The online version contains supplementary material available at 10.1186/s13023-024-03247-1.

## Background

Hereditary angioedema (HAE) is a rare, debilitating, and potentially life-threatening autosomal dominant disease [1, 2]. The majority of patients with HAE have either a deficiency in C1-esterase inhibitor (type I) or dysfunctional C1-esterase inhibitor (type II), resulting in dysregulation of the kallikrein–kinin pathway and overproduction of bradykinin, which leads to unpredictable episodes of increased vascular permeability, extravasation, and subsequent angioedema attacks [2–4]. These intermittent attacks involve cutaneous and mucosal swellings that may affect the extremities, face, larynx, gastrointestinal tract, or genitourinary area [2]. Abdominal symptoms including pain, nausea, diarrhea, and vomiting occur frequently [1]. Laryngeal swelling occurs infrequently but makes asphyxiation an omnipresent risk for patients with HAE [5, 6].

The disease burden extends beyond the frequency and severity of attacks, as a result of interference with activities of daily living, emotional disturbance, and the need for lifestyle modification. Physical functioning may be limited during attacks, restricting participation in work and social activities [7]. Anxiety and depression are common between attacks, due to fear of the next attack and uncertainty regarding the severity of future attacks [8]. Thus, HAE interferes with health-related quality of life (HRQoL) both during and between attacks [7]. Several recent publications have noted the overall disease burden and adverse effects on quality of life associated with HAE and the unmet need for more effective treatment [9, 10].

According to international (World Allergy Organization/European Academy of Allergy and Clinical Immunology) guidelines, the goals of treatment of HAE include achieving complete control of the disease, which for patients means absence of all attacks and normalization of quality of life; the guidelines stipulate that this can only be realized with long-term prophylaxis (LTP) [11]. Hence, LTP is a critical part of pharmacological care for patients with HAE [2, 11]. Treatment guidelines from the US Hereditary Angioedema Association (HAEA) advisory board also recognize that further advancements with novel long-term prophylactic treatment options for HAE (namely C1-esterase inhibitor SC [human], lanadelumab, and berotralstat) have led to a shift in the paradigm of care, with the ability to achieve the realistic treatment targets of reducing the frequency and severity of attacks [2].

C1-esterase inhibitor SC (human), a plasma-derived concentrate of C1-esterase inhibitor (human) for subcutaneous administration twice weekly [12]; lanadelumab, a human monoclonal antibody inhibitor of plasma kallikrein administered subcutaneously once every 2 or 4 weeks [13]; and berotralstat, an oral plasma kallikrein inhibitor administered once daily [14], are currently recommended as first-line LTP in the international guidelines [11]. All three agents provide protection from HAE attacks versus placebo in randomized controlled trials, with sustained long-term prophylactic effects [13, 15–17]. By comparison, LTP available prior to the introduction of these agents mainly consisted of attenuated androgens and intravenous C1 inhibitor concentrate administered twice weekly. Although androgens do prevent HAE attacks, they are commonly associated with side effects including virilization, menstrual disorders, and amenorrhea in women, as well as weight gain, headaches, myalgia, depression, and drug interactions [11, 18]. Treatment with intravenous C1 inhibitor concentrate was burdensome and often led to vein fatigue [19]. Patients with HAE not taking LTP have typically relied on on-demand therapy when an HAE attack occurs [2, 11].

In Phase 3 clinical studies of these novel agents, the reported reduction in HAE attacks and the proportion of HAE patients who were attack free were different for different agents, which might be due to differences in study designs, patient populations, and durations of follow-up. There was a 95.1% median reduction in HAE attacks compared with placebo over 16 weeks in the Phase 3 study for C1-esterase inhibitor SC (human) based on the recommended dose of 60 IU, with 40% attack free during this time [16]. In the 26-week pivotal Phase 3 lanadelumab study, the mean reductions in HAE attacks were 87% or 73% compared with placebo for the recommended 300 mg dose given every 2 or 4 weeks, respectively. Approximately 44% or 31% of patients on the 300 mg dose given every 2 or 4 weeks, respectively, were attack free during the 26-week treatment period, compared with 2.4% of placebo-treated patients [20]. For berotralstat, in the pivotal Phase 3 study at the recommended dose of 150 mg daily, the mean reduction in HAE attacks compared with placebo was 44.2% over 24 weeks [17]. The proportions of patients attack free over the 24-week treatment period were no different in the berotralstat- and placebo-treated groups [17]. A recent real-world study found that use of novel LTP was associated with a 77% reduction in the number of attacks each year compared with those who used only on-demand treatment [1].

Older agents such as attenuated androgens and antifibrinolytic agents are recommended as second-line LTP, for use only when first-line medications are not available. With the availability of novel LTP with greater efficacy and fewer adverse effects compared with androgen therapy, guidelines are focused on reducing the burden of illness due to HAE, which can be achieved by further reducing the frequency of HAE attacks and enabling patients with HAE to experience the same HRQoL as people without this condition [2, 11].

Several disease-specific and generic patient-reported outcome tools are available to evaluate HRQoL for HAE. The Angioedema Quality of Life Questionnaire (AE-QoL) [21, 22] was the disease-specific patient-reported outcome measure included in the development programs for all three novel LTP therapies [8, 23–25]. Although not specific for HAE, it has been validated as a reliable instrument for measuring HRQoL in adult patients with HAE [26]. Reductions in HAE attacks mean that patients may be attack free for more time, which has the potential to improve their HRQoL. A survey of 737 patients with HAE who were members of the US HAEA, conducted by Castaldo et al. [1], showed that median AE-QoL scores for those who had been attack free for 3 months were better than for those who were attack free for only 1 month before completing the AE-QoL. Those on any LTP experienced better QoL than those using only on-demand treatment. In the clinical development programs for the novel prophylactic agents, the relationship between AE-QoL scores and time attack free was not reported in any of the studies apart for an open-label extension study for lanadelumab [20], which showed that most of the improvements in AE-QoL scores were observed during the early follow-up period (day 0 to day 56) and then reached a plateau; the scores were maintained during subsequent visits.

Here, we sought to further build on the earlier research of Castaldo et al. [1] and evaluate the real-world benefits of LTP over a longer time period. Jean-Baptiste et al. have recently evaluated the symptom experience of HAE patients and concluded that more research is needed on the effect of longer attack-free durations on HRQoL [27]. Using data drawn from a wide-ranging survey of members of the US HAEA and Hereditary Angioedema International (HAEi), we examined the relationship between duration of time attack free, and HRQoL, exploring the possibility that there is an association between observed improvement in HRQoL and attack-free duration.

## Methods

### Study design and patients

This survey was conducted in collaboration with the HAEA and HAEi. A global invitation to participate was provided via electronic mail to their membership and social media (the private Facebook Inc., California, United States, page of the HAEA [28] and HAEi membership) with a link to an online, self-administered survey. Patients with HAE in the US, Australia, Canada, the UK, Germany, and Japan were included. Participants signed an online consent form and received an honorarium. They had the right to withdraw consent, access and receive a copy of data they provided, have all their data erased, and rectify any inaccurate information. A total of 159 patients with a confirmed self-reported diagnosis of HAE, aged ≥ 18 years, and treated with LTP participated. No further inclusion or exclusion criteria were defined for study participation. Institutional review board approval was not sought because the survey responses were anonymous, and data collected could not be linked to the respondent. Responses were collected from October 2021 through April 2022.

### Survey

The survey was conducted by MarketCast International, an independent market research company. It consisted of the AE-QoL and the Angioedema Control Test, as well as 52 questions about the sociodemographic characteristics of the patients, their medical history, and how HAE affected their HRQoL before and after starting LTP. Specifically, the questions captured the frequency and severity of HAE attacks; the amount of rescue medication used, refilled, and kept on hand; the perceived control of HAE; and the impact of LTP on overall HRQoL. (Full questionnaire provided in Supplementary information.) The AE-QoL consists of 17 questions with five response options (never, rarely, occasionally, often, and very often) concerning four domains (Functioning, Fatigue/Mood, Fear/Shame, and Nutrition) [22]. The AE-QoL total score is created through a linear transformation of the raw values of all domains ranging from 0 (minimum) to 100 (maximum), with higher scores indicating greater QoL impairment.

### Statistical analysis

The survey participants were divided into groups according to their reported attack-free duration at the time of the survey: 0– < 1 month, 1– < 6 months, and ≥ 6 months. For these three groups, mean AE-QoL total and domain scores were calculated based on the amount of time survey participants were attack free, regardless of the type of LTP used. The mean number of doses of rescue medication used and quality of life benefits were also explored among the three attack-free duration groups.

Analysis of covariance (ANCOVA) was used to explore the relationship between attack-free duration, type of LTP, and AE-QoL total scores. Type of LTP was categorized as novel (guideline-recommended first-line LTP: C1-esterase inhibitor SC [human], lanadelumab, or berotralstat) or non-novel (any other LTP). One ANCOVA model evaluated the correlation between type of treatment (novel or non-novel) and AE-QoL; an additional ANCOVA model controlled for the attack-free duration. Both ANCOVA models had forced inclusion of the frequency of attacks before starting prophylaxis.

## Results

### Sociodemographic characteristics

In total, 159 survey participants with HAE completed the questionnaire, all of whom reported the type of HAE with which they had been diagnosed. One hundred and eighteen patients (74%) were diagnosed with HAE-C1INH-Type I, 28 (18%) with HAE-C1INH-Type II, and 13 (8%) with HAE-nC1INH. The duration of time they had been attack free at the time of the survey was also reported, in response to the question “How long has it been since your last HAE attack?” Seventy-one patients (45%) had been attack free for 0– < 1 month, 43 (27%) for 1– < 6 months, and 45 (28%) for ≥ 6 months at the time of completing the survey. The sociodemographic characteristics of the participants are presented in Table 1. The mean age of the participants was 44.9 years (standard deviation [SD] 13.8) and 79% were female. Eighty-five (54%) of the survey participants were from the US and 73 (46%) from elsewhere.

**Table 1:** Patient demographic characteristics, medical history, perception of HAE health status, and rescue medication patterns prior to starting LTP

Parameter	Total (*N* = 159)
*Patient demographics*	
Geographic location, n (%)	
United States	85 (54)
Outside of the United States	73 (46)
Australia	25 (16)
Canada	7 (4)
United Kingdom	20 (13)
Germany	18 (11)
Japan	4 (2)
Mean age, years (SD)^a^	44.9 (13.8)
Female, n (%)	126 (79.2)
*HAE medical history*	
Mean time since HAE diagnosis, years (SD)	25.0 (13.9)
Mean time since starting prophylactic treatment, years (SD)	10.4 (10.9)
Mean attack frequency/month prior to LTP (SD)	7.13 (7.0)
*Perception of HAE health status*	
HAE attacks were mostly severe prior to LTP, n (%)	118 (74)
Often or always felt anxious about the next attack prior to LTP, n (%)	134 (84)
Overall quality of life was fair or poor prior to LTP, n (%)	129 (81)
HAE perceived as “not at all controlled” prior to LTP, n (%)	103 (65)
*Rescue medication*	
Using on-demand medication prior to LTP, n (%)	110 (69)
Mean number of rescue medication doses per month prior to starting LTP (SD)	
Keep on hand	6.0 (7.9)
Use	4.7 (6.5)
Refill/replace	4.7 (7.1)

^a^Based on non-missing responses from 154 participants

### HAE medical history, perceptions regarding HAE status, and rescue medication consumption prior to use of prophylactic therapy

Table 1 also provides a summary of the HAE medical history of the survey participants, their perception of their HAE status at the time of the survey, and rescue medication use prior to starting LTP. The average time since initial HAE diagnosis was 25.0 years (SD 13.9) and the average time since starting any LTP to treat HAE was 10.4 years (SD 10.9). Overall QoL was reported to be fair or poor in 81% of survey participants, and 69% used on-demand medication prior to starting LTP.

### Goals of LTP reported and perceptions regarding HAE status after starting LTP

Survey participants were asked to describe their personal goals for LTP at the time of starting prophylaxis: 87% reported that they wanted to reduce the frequency of their HAE attacks, 75% wanted to reduce attack severity, 70% wanted to reduce or eliminate the most troublesome attack symptoms, 68% wanted to be attack free, 49% wanted to reduce the psychological problems associated with HAE, and 39% wanted to reduce the amount of on-demand medication they were using.

Table 2 summarizes the pattern of attacks after starting LTP. The average number of attacks per month was 3.0 (SD 5.0) (compared with 7.13 [SD 7.0] before starting LTP) and 31% of participants described themselves as attack free on their current LTP.

**Table 2:** Pattern of attacks after starting LTP

Parameter	Total (*N* = 159)
< 1 month since last attack, n (%)^a^	71 (45)
Mean time since last attack, months (SD)	7.6 (15.6)
Mean number of attacks per month (SD)^b^	3.0 (5.0)
Range	0, 30.4
Describe self as “attack free”, n (%)	50 (31)

^a^Based on responses from all 159 participants

^b^Based on non-missing responses from 109 patients to the question “How frequently do you have attacks while on your current long-term prophylactic medication?”

Patients’ perceived HAE statuses are summarized in Table 3 by duration of time attack free after starting any LTP, showing that the benefits of LTP extend beyond simply reduced attack frequency and severity. After starting LTP, regardless of type, 70% stated that it had reduced or eliminated the most troublesome attack symptoms, and such experience was reported by 87% of the participants who had been attack free for ≥ 6 months. Patients who had been attack free for ≥ 6 months at the time of the survey consistently reported the greatest benefits, characterized by less anxiety and fear about having attacks, fewer HAE-related psychological problems, and less time missed from school/work. Of those who had been attack free for ≥ 6 months, 89% reported that they had less anxiety/fear about having an attack, compared with 49% of those attack free for < 1 month. Sixty-two percent of those attack free for ≥ 6 months reported fewer days missed from school or work compared with 46% of those attack free for < 1 month. Corresponding percentages for not needing to limit social and/or physical activity were 60% and 22%, respectively.

**Table 3:** Quality of life benefits according to attack-free duration after starting LTP

	**Attack-free duration**			
**Quality of life benefits, n (%)**	**All** ***N* = 155**^a^ **N (%)**	**0– < 1 month** ***n* = 67** **n (%)**	**1– < 6 months** ***n* = 43** **n (%)**	** ≥ 6 months** ***n* = 45** **n (%)**
It has reduced/eliminated the most troublesome attack symptoms	108 (70)	39 (58)	30 (70)	39 (87)
It has reduced attack frequency	132 (85)	54 (81)	38 (88)	40 (89)
It has reduced attack severity	106 (68)	48 (72)	26 (60)	32 (71)
It has reduced anxiety/fear of having an attack	101 (65)	33 (49)	28 (65)	40 (89)
It has reduced hospitalization due to attacks	83 (54)	39 (58)	22 (51)	22 (49)
It has reduced psychological problems associated with HAE (stress/fear/anxiety/depression etc.)	63 (41)	19 (28)	19 (44)	25 (56)
It has reduced the amount of HAE medication I was taking	63 (41)	19 (28)	20 (47)	24 (53)
It has reduced the frequency of taking HAE medications	66 (43)	19 (28)	19 (44)	28 (62)
It has reduced anxiety/fear in relation to work/school and social/leisure activities	80 (52)	30 (45)	19 (44)	31 (69)
It has reduced the number of days missed from school/work	75 (48)	31 (46)	16 (37)	28 (62)
I am able to sleep better	36 (23)	13 (19)	8 (19)	15 (33)
I do not need to limit my social and/or physical activity	59 (38)	15 (22)	17 (40)	27 (60)
Other	8 (5)	3 (4)	4 (9)	1 (2)

^a^Excludes four subjects who did not respond to this question

### Relationship between attack-free duration and mean AE-QoL total and domain scores

**Fig. 1:**
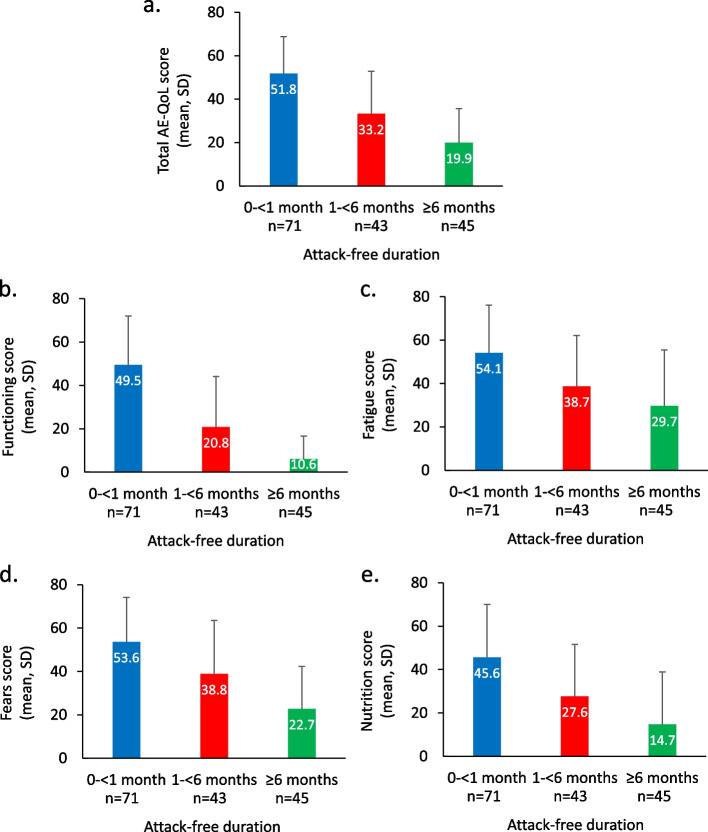
**a**-**e** Relationship between attack-free duration and mean AE-QoL total and individual domain scores, mean and standard deviation (SD), regardless of treatment type (lower scores indicate less QoL impairment). **a** Mean AE-QoL total score; **b** Mean AE-QoL score, Functioning domain; **c** Mean AE-QoL score, Fatigue/Mood domain; **d** Mean AE-QoL score, Fear/Shame domain; **e** Mean AE-QoL score, Nutrition domain

Figure 1a shows the relationship between attack-free duration and mean AE-QoL, regardless of treatment regimen (lower scores indicate less QoL impairment). Compared with those who had been attack free < 1 month (AE-QoL mean [SD], 51.8 [17.0]), mean AE-QoL scores were lower for those attack free for 1– < 6 months (AE-QoL mean [SD], 33.2 [19.7]), and even lower for those attack free for ≥ 6 months (AE-QoL mean [SD], 19.9 [15.9]). The scores for each of the four individual AE-QoL domains followed a similar pattern (Fig. 1b–e) and were consistent with the quality-of-life benefits reported in Table 3. The Functioning domain showed the greatest extent of better scores < 1 month and ≥ 6 months attack-free duration, with the mean scores being 49.5 to 10.6, respectively.

### Relationship between duration of attack-free period and rescue medications used

Use of rescue medication according to attack-free duration at the time of the survey is shown in Table 4. Frequency of rescue medication use was lower for participants with longer attack-free durations: all those attack free for ≥ 6 months reported using rescue medication no more than once per month, compared with 48% of those attack free for < 1 month. Of those attack free for < 1 month, 32% used rescue medication more than twice per month and 6% used it > 10 times per month. Interestingly, of those attack free for ≥ 6 months, 56% report use of rescue medications > 0–1 times per month, indicating continued, but limited, use of rescue medications.

**Table 4:** Frequency of rescue medication usage according to time attack free after starting LTP

Rescue medication usage per month	Attack-free duration			
	**All** ***n* = 159** **N (%)**	**0– < 1 month** ***n* = 71** **n (%)**	**1– < 6 months** ***n* = 43** **n (%)**	** ≥ 6 months *n* = 45** **n (%)**
0	35 (22)	8 (11)	7 (17)	20 (44)
> 0–1	85 (53)	26 (37)	34 (79)	25 (56)
> 1–2	15 (9)	15 (21)	0	0
> 2–5	14 (9)	14 (20)	0	0
> 5–10	5 (3)	4 (6)	1 (2)	0
> 10	5 (3)	4 (6)	1 (2)	0

### Relationship between type of LTP, duration of attack-free period, and AE-QoL score

The mean AE-QoL score was lower in patients treated with novel LTP compared with those receiving non-novel LTP (Figure S1 in Additional material). In order to evaluate the relationships between type of LTP treatment used, attack-free duration, and AE-QoL, we applied ANCOVA to model results according to type of LTP: novel (*n* = 112, C1-esterase inhibitor SC [human], lanadelumab, or berotralstat) versus non-novel (*n* = 47, any other LTP, which included C1-esterase inhibitor intravenous [IV]). Estimate and p-value (Pr >|t|) of each effect from the ANCOVA model illustrated the magnitude and strength of the association with AE-QoL. The larger estimate along with smaller *p*-value displayed the larger and stronger association of the effect with AE-QoL. The ANCOVA in Table 5, without consideration of attack-free duration, showed a strong correlation between type of LTP (novel versus non-novel) and the AE-QoL total score, with novel LTP being associated with better AE-QoL (Pr > [t] < 0.0001). When controlling for attack-free duration in categories of 0– < 1 month, 1– < 6 months, and ≥ 6 months, the other ANCOVA in Table 6 showed that the association between type of treatment and AE-QoL score was reduced; the corresponding estimate for type of LTP is 9.0 (*p* < 0.0032) in Table 6 versus 15.8 (*p* < 0.0001) in Table 5 without such control. Thus, the duration of the attack-free period had a strong association with AE-QoL aligned with the results shown in Fig. 1.

**Table 5:** ANCOVA showing correlation between type of treatment and AE-QoL

Effect	Group	t-value	Pr > [t]	Estimate	Standard error	DF
Intercept		9.8	< 0.0001	24.6	2.5	156
Type of LTP	Non-novel	4.6	< 0.0001	15.8	3.4	156
	Novel	-	-	0	-	-
Monthly attack rate prior to LTP		5.3	< 0.0001	1.2	0.2	156

*DF* degrees of freedom, Pr > [t], the p-value associated with t

**Table 6:** ANCOVA showing correlation between type of treatment, attack-free duration, and AE-QoL

Effect	Group	t-value	Pr > [t]	Estimate	Standard error	DF
Intercept		5.3	< 0.0001	14.6	2.7	154
Type of LTP	Non-novel	3.0	0.0032	9.0	3.0	154
	Novel	-	-	0	-	-
Attack-free duration	0– < 1 month	7.9	< 0.0001	26.6	3.4	154
	1– < 6 months	3.3	0.0012	11.7	3.6	154
	≥ 6 months	-	-	0	-	-
Monthly attack rate prior to LTP		3.8	0.0002	0.8	0.2	154

*DF* degrees of freedom, Pr > [t], the p-value associated with t

## Discussion

This study reports the results from an international survey of patients with HAE who are currently on LTP and are members of the HAEA or the HAEi, conducted in the US, Australia, Germany, Canada, the UK, and Japan. The survey included the AE-QoL as well as 52 questions related to HAE medical history and the impact of HAE on participants’ HRQoL before and after receiving LTP. The analysis reported here was designed to assess the importance of longer attack-free time at the time of the survey through its relationship to better reported HRQoL.

In recent years, several published studies have shown reductions in HAE attacks with the use of novel prophylactic regimens [16, 17, 20, 29], allowing patients with HAE to be attack free longer. Despite this, only one study has examined the relationship between attack-free time and HRQoL. Castaldo et al. [1] reported that, for those receiving any LTP, the median AE-QoL total score was 25.7 for those who had been attack free for 1 month before completing the AE-QoL compared with 20.6 for those attack free for 3 months. The results reported here extend that research by including patients who reported being attack free for longer periods of time and examining the association of attack-free duration with AE-QoL scores and several HAE status factors (including attack frequency and severity, psychological status, and everyday activities). The association between being attack free and the number of doses of rescue medication used is also reported.

The benefits of a longer attack-free duration include less anxiety/fear of having an attack, fewer psychological problems, fewer days missed from school or work, and fewer limitations on social and/or physical activity. The mean AE-QoL scores improved from 51.8 in those attack free for < 1 month to 19.9 in those attack-free for ≥ 6 months. These benefits of LTP for HRQoL are consistent with the goals of therapy reported by the survey participants, which include lower attack frequency and severity, fewer of the most troublesome attack symptoms, and fewer psychological problems associated with HAE. These findings are consistent with a recent real-world study showing that LTP with a novel agent contributes to the goal of normalizing patients’ lives by reducing the frequency and severity of attacks and improving quality of life [30]. Both US and international guidelines emphasize that the introduction of novel LTP has resulted in a paradigm shift, due to their greater efficacy in reducing HAE attacks, better tolerability, and beneficial effect on quality of life compared with earlier therapies [2, 11]. The ANCOVA models showed a correlation between the use of novel LTP and better AE-QoL score but the strength of association was reduced after controlling for attack-free duration, indicating that the longer attack-free time associated with the use of novel LTP had an influential role for the better AE-QoL total scores. Increasing the attack-free period is an important goal of therapy and can be achieved with currently available first-line LTP with novel agents. However, findings from this survey highlight that opportunities still exist to improve the treatment impact on attack free duration and HRQoL if new therapies are approved that allow more people to be attack free for longer time periods. Recent publications also emphasize the enduring disease burden and adverse impact of HAE on quality of life despite the availability of novel LTP [31, 32].

Less use of rescue medication was also associated with longer attack-free durations: all those attack free for ≥ 6 months reported using no more than one dose per month, compared with only 48% of those attack free for < 1 month; conversely, 12% of those attack free for < 1 month used more than five doses/month.

Several additional aspects of the survey results deserve further discussion, including some apparent inconsistencies. It should be acknowledged that the responses reflect patients’ experience and perception. Prior to starting LTP, 65% reported that their HAE was not at all controlled, while 74% indicated that their attacks were mostly severe, and 84% were always or often anxious about the next attack. These potential contradictions might be attributable to the way the survey questions were phrased and the respondents’ understanding of control. Other possible response options to the question “How well controlled would you say your HAE was prior to taking any LTP” were “a little,” “somewhat,” “well,” and “very well,” so the remaining 35% believed their HAE was at least partially controlled. Only 69% of patients reported using rescue medication prior to starting LTP, which may have had an adverse effect on their quality of life.

Somewhat surprisingly, only 41% of all respondents stated that they experienced fewer psychological problems after starting LTP, although this was higher at 56% in those who had been attack free for ≥ 6 months. Nevertheless, the proportion feeling less anxious was higher and is likely to continue to be better with longer treatment, as indicated by 84% reporting that they would have less anxiety about having an attack the longer they remained attack free. It may also reflect the impact of adverse effects in those on older, non-novel LTP.

Interestingly, only 54% of respondents reported less hospitalization due to HAE attacks since starting LTP (49% in those attack free for ≥ 6 months), even though 85% reported lower attack frequency. This may seem lower than expected but given that a significant proportion of patients may not have had any hospitalization prior to starting LTP, these patients would not have been able to report a reduction in it. Similarly, despite reporting lower attack frequency and severity, only 43% indicated that they used less rescue medication (62% in those attack free for ≥ 6 months). It should be noted that guidelines state that it is essential that patients have on-demand (rescue) medication available to treat all attacks as early as possible [11].

This study has certain weaknesses and limitations. The diagnosis of HAE was self-reported and was not verified. Respondents were asked about their use of medication, but it is possible that not all drugs potentially used for LTP were available in every country at the time of the survey. There is the potential for recall bias when asking survey participants about the impact of HAE many years ago, before starting prophylactic therapy, which may limit our ability to accurately interpret the experiences of the survey participants before and after starting LTP. As this is an observational study in a real-world setting, the analyses may not have accounted for all the differences between groups. This could have introduced bias, especially if other factors without control in the model were associated with such differences.

Despite these limitations, the study adds important new information on the broader role of LTP beyond the physical effect of reducing attack frequency and severity. It describes the benefit of being attack free for specific time periods with respect to the amount of rescue medication used. Longer reported attack-free time at the time of the survey is associated with better AE-QoL total scores and other quality of life benefits, as well as fewer doses of rescue medication used. Most importantly, this study shows that the association of LTP with better HRQoL is substantially influenced by longer attack-free time, thereby supporting the importance of having treatments available that may enable patients to have an extended time free of attacks.

### Supplementary Information

Supplementary Material 1.

Supplementary Material 2.

## Data Availability

The datasets generated and/or analyzed during the current study are not publicly available; CSL Behring will consider on a case-by-case basis requests to share these datasets. Any requests should be made to the corresponding author.

## Abbreviations

AE-QoL: Angioedema Quality of Life Questionnaire
ANCOVA: Analysis of covariance
HAE: Hereditary angioedema
HAEA: US Hereditary Angioedema Association
HAEi: Hereditary Angioedema International
HRQoL: Health-related quality of life
LTP: Long-term prophylaxis
QoL: Quality of life
