# Supplementary material for: An international survey assessing the effects of the duration of attack-free period on health-related quality of life for patients with hereditary angioedema
Source: Orphanet J Rare Dis. 2024 Jun 22;19:241. doi: 10.1186/s13023-024-03247-1 (PMC11193256; doi:10.1186/s13023-024-03247-1)
Supplement: Supplementary file 1 — Supplementary Material 1. [file 13023_2024_3247_MOESM1_ESM.pdf]

HAE Patient Characteristics and Patient Reported Outcome

Thank you for entering this survey. It is being conducted by Phoenix Marketing International, an independent market research company.
We are currently conducting research on the subject of Hereditary Angioedema (HAE) and would like to understand your views and experience. First if you could please answer a few questions to find out if this study is of relevance to you.

S1	What type of HAE are you diagnosed with?<br>*Please select one*		
	Type I HAE	1	**CONTINUE TO S2a**
	Type II HAE	2	
	Type III HAE/ nC1-INH-HAE	3	
	Don't know	4	**CLOSE**
	Not diagnosed with HAE		**CLOSE**

S2a	Are you aged 18 years or older?<br>*Please select one*		
	Yes	1	**CONTINUE TO S2b**
	No	2	**CLOSE**

S2b	How old are you?		
	[__][__][__] age in years		**CONTINUE TO S3**
	Prefer not to give my age	2	

S3	Do you use <u>long term prophylactic medications</u> regularly to prevent HAE attacks? This means you take medication **on a regular, <u>long-term</u> basis**, as prescribed by your physician, to prevent HAE symptoms instead of starting medication after the HAE attacks begin or when you experience prodromal symptoms (i.e. any warning signs or symptoms that signal you are going to have an attack)	
	Yes	**CONTINUE TO S4**
	No	**CLOSE**

S4	Do you currently work as a consultant or employee, in any capacity, for a pharmaceutical company?<br>*Please select one*		
	Yes	1	**CLOSE**
	No	2	**CONTINUE TO CONSENT**

Thank you.
Based on your responses, we are very pleased to invite you to take part in our survey. Please click the advance button to enter the survey

Consent for Personal Information Capture and Data Transfer

Phoenix Marketing International, (PMI) a major international research services provider, is conducting research among HAE patients. As a participant in this survey you will not be asked to provide any personal information to Phoenix Marketing International

Neither the results of this research, or potential subsequent publication of the results, will identify you individually in any way, and will only be a summation of all participants' responses. Your personally identifiable information will not be linked directly to any responses.

Specifics

All data collected will be controlled by Phoenix Marketing International in the United States for the purpose of generating research and analysis for our client. We are also aiming to publish the results of our research in a recognized medical journal.

PMI maintains the highest levels of data security and is EU-US Privacy Shield and ISO 27001 Certified. Our privacy statement can be found at https://phoenixmi.com/privacy-policy/

If at any time you have concerns or questions you can contact our Chief Privacy and Data Protection Officer (DPO) at

Phoenix Marketing International
c/o Chief Privacy and Data Protection Officer
6423 Montgomery Street, Suite 12
Rhinebeck, NY 12572
Phone: 845-876-8228
Fax: 845-876-8284
Email: privacy@phoenixmi.com

The DPO can assist you with:

Your Right to:

1. withdraw consent
2. access and receive a copy of the data you have provided
3. have PMI erase all data we collected from your participation
4. rectification of any inaccurate information

By checking the consent box below you indicate that you have read and understand how we will be utilizing the information you provide, along with your rights as a participant.

If you check the "I do not consent" box, you will not be able to continue with the survey

Please select one

I provide my consent for Phoenix Marketing International to utilize that information for the indicated purpose, and transfer that information as detailed above	1	**CONTINUE TO ADVERSE EVENTS**
I do not consent	2	**CLOSE**

ADVERSE EVENTS

Different patients sometimes respond in different ways to the same medicine, and some side effects may not be discovered until many people have used a medicine over a period of time. For this reason, we are now required to pass on to our client, who is a manufacturer of medicines, details of any side effects/product complaints related to their own products that are mentioned during the course of market research.

Although the responses you give during the survey will, of course, be treated in confidence, should we become aware of a side effect/product complaint when you, the person you care for or someone you know, became ill after taking one of our client's medicines, we will need to report this, so they can learn more about the safety of their medicines.

If you agree to waive confidentiality, your name and contact details will be forwarded to the sponsor's Pharmacovigilance department for the express and sole purpose of follow-up of such report(s). All other information that you give us in the context of this market research will continue to remain confidential.

If you prefer to preserve your confidentiality regarding any adverse event reporting, please select 'I do not agree'. This will not cause you to be terminated from the study

Please select one

I agree	1	**CONTINUE TO QUESTIONNAIRE**
I do not agree	2	

Questionnaire

TERMINOLOGY

Please see below for explanations of specific terms which you will see when going through the survey. There will be link throughout the survey that you can click on to remind yourself of the definitions if necessary
INSERT LINK THROUGHOUT QUESTIONNAIRE

- The following terms are generally used to describe what most consider to be an HAE attack: “angioedema,” “attack,” “HAE attack,” “swelling episode.”
- If only “swelling” or “symptoms” are mentioned, this would <u>include</u> swelling or symptoms that occur during what you consider to be an “HAE attack” but would <u>also include</u> **either signs or symptoms** that may suggest an attack may be coming on, **or symptoms** that you would not consider to be an actual “HAE attack” but which would still be noticeable or troublesome.
- <u>Long term prophylactic medication</u> = medication you take on a **regular** basis, (regardless of whether you have an attack), which is intended to **prevent** HAE symptoms
- <u>Rescue medication</u> - medication that you take when you are having an attack or feel symptoms or signs that suggest an attack may be coming on

AECT questions

Before we go into the main section of the questionnaire please could you answer these two sets of questions about how well controlled your HAE is at present.

Qi	In the last 3 months, how often have you had angioedema?<br>*Select one*	
	Very often	1
	Often	2
	Sometimes	3
	Seldom	4
	Not at all	5

Qii	In the last 3 months, how much has your quality of life been affected by angioedema?<br>*Select one*	
	Very much	1
	Much	2
	Somewhat	3
	A little	4
	Not at all	5

Qiii	In the last 3 months, how much has the unpredictability of your angioedema bothered you?<br>*Select one*	
	Very much	1
	Much	2
	Somewhat	3
	A little	4
	Not at all	5

Qiv	In the last 3 months, how well has your angioedema been controlled by your therapy?<br>*Select one*	
	Not at all	1
	A little	2
	Somewhat	3
	Well	4
	Very well	5

AE-QoL questions

Qa	Indicate how often within the **last 4 weeks** you have been restricted in the areas of your daily life listed below because of swelling episodes (angioedema). (regardless of whether or not you have actually experienced swelling episodes during that time period)				
		Never	Rarely	Occasionally	Often	Very often
	Work	☐	☐	☐	☐	☐
	Physical activity	☐	☐	☐	☐	☐
	Leisure time	☐	☐	☐	☐	☐
	Social relations	☐	☐	☐	☐	☐
	Eating and drinking	☐	☐	☐	☐	☐

Qb	In the following questions we would like to get more details about the difficulties and problems that may be associated with your recurrent swelling episodes (angioedema) (**during the last 4 weeks**)					
		Never	Rarely	Occasionally	Often	Very often
	Do you have difficulty falling asleep?	☐	☐	☐	☐	☐
	Do you wake up during the night?	☐	☐	☐	☐	☐
	Are you tired during the day because you are not sleeping well at night?	☐	☐	☐	☐	☐
	Do you have trouble concentrating?	☐	☐	☐	☐	☐
	Do you feel depressed?	☐	☐	☐	☐	☐
	Do you have to limit your choices of food or beverages?	☐	☐	☐	☐	☐
	Do the swelling episodes place a burden on you?	☐	☐	☐	☐	☐
	Are you afraid that a swelling episode could occur suddenly?	☐	☐	☐	☐	☐
	Are you afraid that the frequency of the swelling episodes might increase?	☐	☐	☐	☐	☐

	Are you ashamed to go out in public because of the swelling episodes?	☐	☐	☐	☐	☐
	Do the swelling episodes make you embarrassed or self-conscious?	☐	☐	☐	☐	☐
	Are you afraid that the treatment of the swelling episodes could have negative long-term effects for you?					

Main Questionnaire

First, I would like to ask you some general questions about your HAE and how it is treated

Q1	How many years has it been since you were diagnosed with HAE?
	[__][__][__] years

Q2	How long have you been taking any type of **long-term** prophylactic medication for your HAE?
	[__][__][__] days/weeks/months/years<br>*Please enter a number and select a time period*<br>**INSERT PULL DOWN MENU FOR TIME PERIODS**

Q3a	Which long-term prophylactic HAE medications have you ever taken during this time to **prevent** attacks?<br>*(Please select each medication you have ever taken)*	
	IV C1 inhibitor (e.g. Cinryze, Ruconest **[DO NOT SHOW RUCONEST IN THE UK]**, etc.)	1
	Takhzyro (subcutaneous lanadelumab)	2
	Haegarda (subcutaneous C1 inhibitor) **[USA/CANADA NAME]**<br>Berinert 2000/3000 (subcutaneous C1 inhibitor) **[NON USA/CANADA NAME] DO NOT SHOW IN UK**	3
	Orladeyo (oral berotralstat)	4
	Androgens	5
	Other	6

Q4a	Which of the following long-term prophylactic medications are you **currently** taking to **prevent** HAE attacks?<br>*Please select one*<br>**DO NOT ASK IF ONLY ONE MEDICATION LISTED IN Q3a,**<br>**IF MORE THAN ONE SELECTED IN Q3a, PIPE IN EVER USED MEDICATION LIST**	
	IV C1 inhibitor (e.g. Cinryze, Ruconest **[DO NOT SHOW RUCONEST IN THE UK]**, etc.)	1
	Takhzyro (subcutaneous lanadelumab)	2
	Haegarda (subcutaneous C1 inhibitor) **[USA/CANADA NAME]**<br>Berinert 2000/3000 (subcutaneous C1 inhibitor) **[NON USA/CANADA NAME] DO NOT SHOW IN UK**	3
	Orladeyo (oral berotralstat)	4
	Androgens	5

	Other	6

Q4b	**IF CODE 1 SELECTED AT Q4a**<br>Which specific IV C1 inhibitor are **currently** taking to **prevent** HAE attacks?<br>*Please select one*	
	Cinryze (IV C1 inhibitor)	1
	Berinert (IV C1 inhibitor) **[USA/CANADA NAME]**<br>Berinert 500/1500 (IV C1 inhibitor) **[NON USA/CANADA NAME]**<br>**DO NOT SHOW IN UK**	2
	Ruconest (IV C1 inhibitor)	3
	Other/Don't know	4

Q5	And how long have you been taking **[PIPE IN RESPONSE FROM Q4a OR Q4b]** as your **current** long-term prophylactic medication for your HAE?
	[__][__][__] days/weeks/months/years<br>*Please enter a number and select a time period*<br>**INSERT PULL DOWN MENU FOR TIME PERIODS**

Q6	Do you take **[PIPE IN RESPONSE FROM Q4a OR Q4b]** regularly as prescribed by your doctor?<br>*Please select one*	
	Yes, I almost always take it exactly as my doctor prescribed	1
	No, I take it on a different schedule, or when I feel like I need it	2

Q7	**IF Q4a = CODE 2 (TAKHZYRO) ASK:**<br>How frequently do you use Takhzyro (subcutaneous lanadelumab)?<br>*Please select one*	
	Once every 2 weeks	1
	Once every 4 weeks	2
	Once every 6 weeks	3
	Once every 8 weeks	4
	Other, please specify<br>_____________	5

Q8	How well controlled is your HAE on your **current** long-term prophylactic medication?<br>*Please select one*

	Not at all controlled	1
	A Little controlled	2
	Somewhat controlled	3
	Well controlled	4
	Very well controlled	5

Q9	On your **current** long-term prophylactic medication, how often are you anxious about your next attack?<br>*Please select one*	
	Never	1
	Rarely	2
	Sometimes	3
	Often	4
	Always	5

Q10	On your **current** long-term prophylactic medication, how often do you take steps to make sure you avoid known triggers for an attack?<br>*Please select one*	
	Rarely	1
	Sometimes	2
	Often	3
	Always	4

Q11	How **satisfied** are you overall with the treatment of your HAE on your **current** long-term prophylactic medication?<br>*Please select one*	
	Extremely dissatisfied	1
	Very dissatisfied	2
	Somewhat satisfied	3
	Satisfied	4
	Very Satisfied	5

Q12	How would you rate your overall quality of life on your **current** long-term prophylactic medication for HAE? *Please select one*	
	Excellent	1
	Very good	2
	Good	3
	Fair	4
	Poor	5

Now we would like to ask you a couple of questions about your rescue medications, that you take when you are having an attack or feel symptoms or signs that suggest an attack may be coming on

Q13	Typically, on your **current** long-term prophylactic medication, how frequently do you use your rescue medication?
	[__][__][__] times per day/week/month/year<br>☐ Never<br>*Please enter a number and select a time period*<br>**INSERT PULL DOWN MENU FOR TIME PERIODS**

Q14	How long is it since you last used your rescue medication?
	[__][__][__] days/weeks/months/years<br>*Please enter a number and select a time period*<br>**INSERT PULL DOWN MENU FOR TIME PERIODS**

Q15	In a typical month, while using your **current** long-term prophylactic medication, how many doses of rescue medication do you: *Please enter a number for each*	
	Keep on hand?	
	Use?	
	Refill/replace?	

Q16	In a typical month **prior to** taking **any** long-term prophylactic medication (when you were only treating acutely with rescue medications), how many doses of rescue medication did you: *Please enter a number for each*	
	Keep on hand?	
	Use?	
	Refill/replace?	

Q17a	Do you ever experience symptoms that you know are HAE related, but which are **unlikely** to cause an attack *Please select one*	
	Yes	1
	No	2

Q17b	**IF YES AT Q17a**<br>Which symptoms do you experience that you know are HAE related, but are **unlikely** to cause an attack?<br>*Please select all which apply*	
	Painless, non-itchy rash	1
	Tingling skin	2
	Skin tightness	3
	Fatigue	4
	Irritability	5
	Mood changes	6
	Anxiety	7
	Depression	8
	PTSD	9
	Other	10

Q17c	**IF YES AT Q17a**<br>How frequently do you experience symptoms that you know are HAE related, but are **unlikely** to cause an attack?
	[__][__][__] times per day/week/month/year<br>*Please enter a number and select a time period*<br>**INSERT PULL DOWN MENU FOR TIME PERIODS**

Q18a	Do you ever experience symptoms that suggest you are going to have an attack?<br>*Please select one*	
	Yes	1
	No	2

Q18b	**IF YES AT Q18a**<br>Which symptoms do you experience symptoms that suggest you are going to have an attack?<br>*Please select all which apply*	
	Painless, non-itchy rash	1
	Tingling skin	2

	Skin tightness	3
	Fatigue	4
	Irritability	5
	Mood changes	6
	Anxiety	7
	Depression	8
	PTSD	9
	Other	10

Q18c	**IF YES AT Q18a**<br>How frequently do you experience symptoms that suggest you are going to have an attack?
	[__][__][__] times per day/week/month/year<br>*Please enter a number and select a time period*<br>**INSERT PULL DOWN MENU FOR TIME PERIODS**

Q18	Currently, if you experience (or were to experience) an HAE attack, which of the following most accurately describes the point at which you start (or would start) to take your rescue medication?<br>*Please select one*<br>**IF NO AT BOTH Q17a AND Q18a SHOW OPTIONS 3 AND 4 ONLY**	
	As soon as I feel any warning signs that I might be about to have an attack (please note by warning signs we mean a signal that an attack is likely to happen, but not necessarily symptoms of the attack itself)	1
	If these early warning signs persist and do not subside	2
	Not until I am sure I am actually having an attack (i.e. swelling)	3
	Once my attack symptoms become disabling	4

Now we are going to ask you some questions about your experiences with HAE while on long-term prophylaxis medication.

Q19	What were your goals when you **first** started taking long-term prophylactic medication for your HAE?<br>*Please select all which apply*	
	Reduce/eliminate the most troublesome attack symptoms	1
	Reduce attack frequency	2
	Reduce attack severity	3
	Be attack free	4

	Reduce hospitalizations due to attacks	5
	Reduce the psychological problems associated with HAE (e.g. stress/fear/ anxiety/depression etc.)	6
	Reduce the amount of on demand medication I was taking	7
	Reduce the frequency of taking on demand medication	8
	Other	9

Q20	To what extent has your quality of life improved since you started taking <u>long-term prophylactic medication</u>?<br>*Please select one*	
	Greatly improved	1
	Somewhat improved	2
	Not at all improved	3

Q21	**IF 1 OR 2 SELECTED AT Q20**<br>And in what way has <u>long-term prophylactic medication</u> led to this improvement?<br>*Please select all which apply*	
	It has reduced/eliminated the most troublesome attack symptoms	1
	It has reduced attack frequency	2
	It has reduced attack severity	3
	It has reduced anxiety/fear of having an attack	4
	It has reduced hospitalization due to attacks	5
	It has reduced psychological problems associated with HAE (e.g. stress/fear/ anxiety/depression etc.)	6
	It has reduced the amount of HAE medication I was taking	7
	It has reduced the frequency of taking HAE medications	8
	It has reduced anxiety/fear in relation to work/school and social/leisure activities	9
	It has reduced the number of days missed from school/work	10
	I am able to sleep better	11
	I do not need to limit my social and/or physical activity	12
	Other	13

And now we would like you to tell us more about your most recent experiences with HAE

Q22	Which of these descriptions matches the **current** <u>overall</u> severity of your HAE most closely?

	Please select one	
	Mild (HAE has little to no effect on my daily activities)	1
	Moderate (HAE causes my daily activities to be difficult)	2
	Severe (HAE causes marked limitations to my daily activities)	3

Q23	How long has it been since your last HAE attack?
	[__][__][__] days/weeks/months/years<br>*Please enter a number and select a time period*<br>**INSERT PULL DOWN MENU FOR TIME PERIODS**

Q24	Do you (or would you) have less anxiety about having attacks the longer you remain attack free?<br>*Please select one*	
	Yes	1
	No	2

Q25	Which of the definitions below most closely matches what you would define as being HAE attack free?<br>*Please select one*	
	Being completely free of all HAE symptoms ,i.e. no swelling ever	1
	Never having warning signs or symptoms severe enough to require rescue medication	2
	Having warning signs or symptoms of an attack which require, and resolve with, rescue medication	3
	Being symptom and swelling free for at least 1 month	4
	Being symptom and swelling free for at least 3 months	5
	Being symptom and swelling free for at least 6 months	6
	Being symptom and swelling free for at least 12 months	7

Q26	How long would you have to be HAE attack free in order to stop worrying about attacks?
	[__][__][__] days/weeks/months/years<br>*Please enter a number and select a time period*<br>**INSERT PULL DOWN MENU FOR TIME PERIODS**

Q27	**ASK IN US ONLY**<br>How long would you need to be HAE attack free in order to reduce the amount of rescue medication you keep on hand?
	[__][__][__] days/weeks/months/years

	☐ I would never reduce the amount of rescue medication<br><br>*Please enter a number and select a time period*<br>**INSERT PULL DOWN MENU FOR TIME PERIODS**

SHOW FOLLOWING SET-UP STATEMENT:

Moving forward, please think of attack-free as being “symptom and swelling free”

Q28	Based upon this definition, would you currently describe yourself as HAE attack free on your **current** long-term prophylactic medication?<br>*Please select one*	
	Yes	1
	No	2

Q29	**IF YES AT Q28**<br>How long have you been HAE attack free based upon this definition?
	[__][__][__] days/weeks/months/years<br><br>*Please enter a number and select a time period*<br>**INSERT PULL DOWN MENU FOR TIME PERIODS**

Q30	**ASK ALL**<br>To what extent do you agree with the definition of HAE Attack Free as being “symptom and swelling free”<br>Please use a 1 – 7 scale where 1 = completely disagree, 4 = neither agree nor disagree, and 7 = completely agree<br>*Please select one*						
	Completely disagree			Neither agree nor disagree			Completely agree
	1	2	3	4	5	6	7

IF RESPONDENT IS ATTACK FREE (CODE 1 at Q28) SKIP TO Q36

Q31	If you are still experiencing attacks on your **current** long-term prophylactic medication, which of the following bodily locations do your attacks generally involve?<br>*Select all that apply*	
	Peripheral (hands, feet, arms and/or legs)	1
	Abdominal	2
	Facial	3
	Laryngeal (i.e. Throat, Airway)	4
	Other	5

Q32	How frequently do you have attacks while on your **current** long-term prophylactic medication?	
	[__][__][__] times per day/week/month/year<br>*Please enter a number and select a time period*<br>**INSERT PULL DOWN MENU FOR TIME PERIODS**	

Q33	And overall, how severe do the HAE attacks tend to be while you are on your **current** long-term prophylactic medication?<br>*Please select one*	
	Attacks are mostly mild (i.e. have little to no effect on my daily activities)	1
	Attacks are mostly moderate (i.e. cause my daily activities to be difficult)	2
	Attacks are mostly severe (i.e. cause marked limitations to my daily activities)	3

Q34	While on your **current** long-term prophylactic medication, do attacks tend to be the same or different in terms of their severity?<br>*Please select one*	
	Same	1
	Different	2

Q35	**ASK IN US ONLY**<br>**IF CODE 2 AT Q28**<br>If you were to be able to be attack free (no symptoms at all for at least six months), how many doses of rescue medication would you then expect to keep on hand in a typical month?
	Please enter number of doses<br>[__][__][__]

Now, please could I ask you to think back to **before** you started taking long-term prophylactic medication to treat your HAE.

Q36	Before you started taking **any** long-term prophylactic medication to treat your HAE, what type of medication did you mainly use? *Please select one*	
	**On-demand** medication, either after an HAE attack began or as soon as you first started to experience any symptoms related to your HAE	1
	**No** medication at all	2

37	Overall, how frequently did you have an HAE attack **prior** to taking **any** long-term prophylactic medication?
	[__][__][__] times per day/week/month/year<br>*(Please enter a number and select a time period)*<br>**INSERT PULL DOWN MENU FOR TIME PERIODS**

Q38	And overall, how severe did your HAE attacks tend to be **prior** to taking **any** long-term prophylactic medication?<br>*Please select one*	
	Attacks were mostly mild (i.e. had little to no effect on my daily activities)	1
	Attacks were mostly moderate (i.e. caused my daily activities to be difficult)	2
	Attacks were mostly severe (i.e. caused marked limitations to my daily activities)	3

Q39	And which of the following bodily locations did your HAE attacks generally involve **prior** to taking **any** long term prophylactic medication?<br>*Select all that apply*	
	Peripheral (hands, feet, arms and/or legs)	1
	Abdominal	2
	Facial	3
	Laryngeal (i.e. Throat, Airway)	4
	Other	5

Q40	What effect did the attacks have on your daily life **prior** to taking **any** long-term prophylactic medication?<br>*Please select all which apply*<br>**RANDOMISE**	
	I was unable to get up/had to stay in bed	1
	I was unable to leave home/had to stay home	2
	I was unable to work/study	3
	I was unable to take part in my usual leisure activities/hobbies	4
	I had increased psychological problems (e.g. fear/anxiety/depression etc.)	5
	My social life was adversely affected	6
	My relations with friends/family/colleagues were adversely affected	7
	I had increased fatigue	8
	I was unable to do my usual household activities (e.g. laundry/ cleaning/ DIY/gardening etc.)	9
	I had to visit more doctors more often	10
	Other	11

Q41	**FOR EACH EFFECT SELECTED in Q40**<br>How did this effect restrict your daily life **prior** to taking **any** long-term prophylactic medication?<br>*Please select one*	
	No restriction	1

	Slight restriction	2
	Severe restriction	3
	No activity possible	4

Q42	**Prior** to taking **any** long-term prophylactic medication, how often were you anxious about your next attack?<br>*Please select one*	
	Never	1
	Rarely	2
	Sometimes	3
	Often	4
	Always	5

Q43	Overall, how well controlled would you say your HAE was **prior** to taking **any** long-term prophylactic medication?<br>*Please select one*	
	Not at all	1
	A Little	2
	Somewhat	3
	Well	4
	Very well	5

Q44	How would you rate your overall quality of life **prior** to taking **any** long-term prophylactic medication?<br>*Please select one*	
	Excellent	1
	Very good	2
	Good	3
	Fair	4
	Poor	5

Q45	Thinking now to the future, what would be your ideal therapy goals which would help you to feel your best?<br>Please indicate how important each of these goals is by allocating 100 points across them. The more points you allocate to a particular goal, the more important you find it	
	Completely symptom free	

	Completely attack free	
	No need to use rescue medication	
	**Total**	**100**

And finally,

Q46	To which gender identity do you most identify?<br>*Please select one*	
	Female	1
	Male	2
	Transgender Female	3
	Transgender Male	4
	Gender Variant/Non-Conforming	5
	Not listed (please enter) ________________	6
	Prefer not to say	7

PROGRAMMER – WHEN RESPONDENT COMPLETES THE SURVEY, ON THE CLOSING PAGE PLEASE HAVE THE PROGRAM GENERATE A UNIQUE CODE FOR EACH RESPONDENT, AND INSERT THE FOLLOWING INSTRUCTION INTO THE CLOSING PAGE

"Thank you for taking part in this survey
Please pass this unique code back to the HAEA and they will use it to organize payment of your compensation"
