# Supplementary material for: An international survey assessing the effects of the duration of attack-free period on health-related quality of life for patients with hereditary angioedema
Source: Orphanet J Rare Dis. 2024 Jun 22;19:241. doi: 10.1186/s13023-024-03247-1 (PMC11193256; doi:10.1186/s13023-024-03247-1)
Supplement: Supplementary file 2 — Supplementary Material 2. [file 13023_2024_3247_MOESM2_ESM.pptx]

## Slide 1
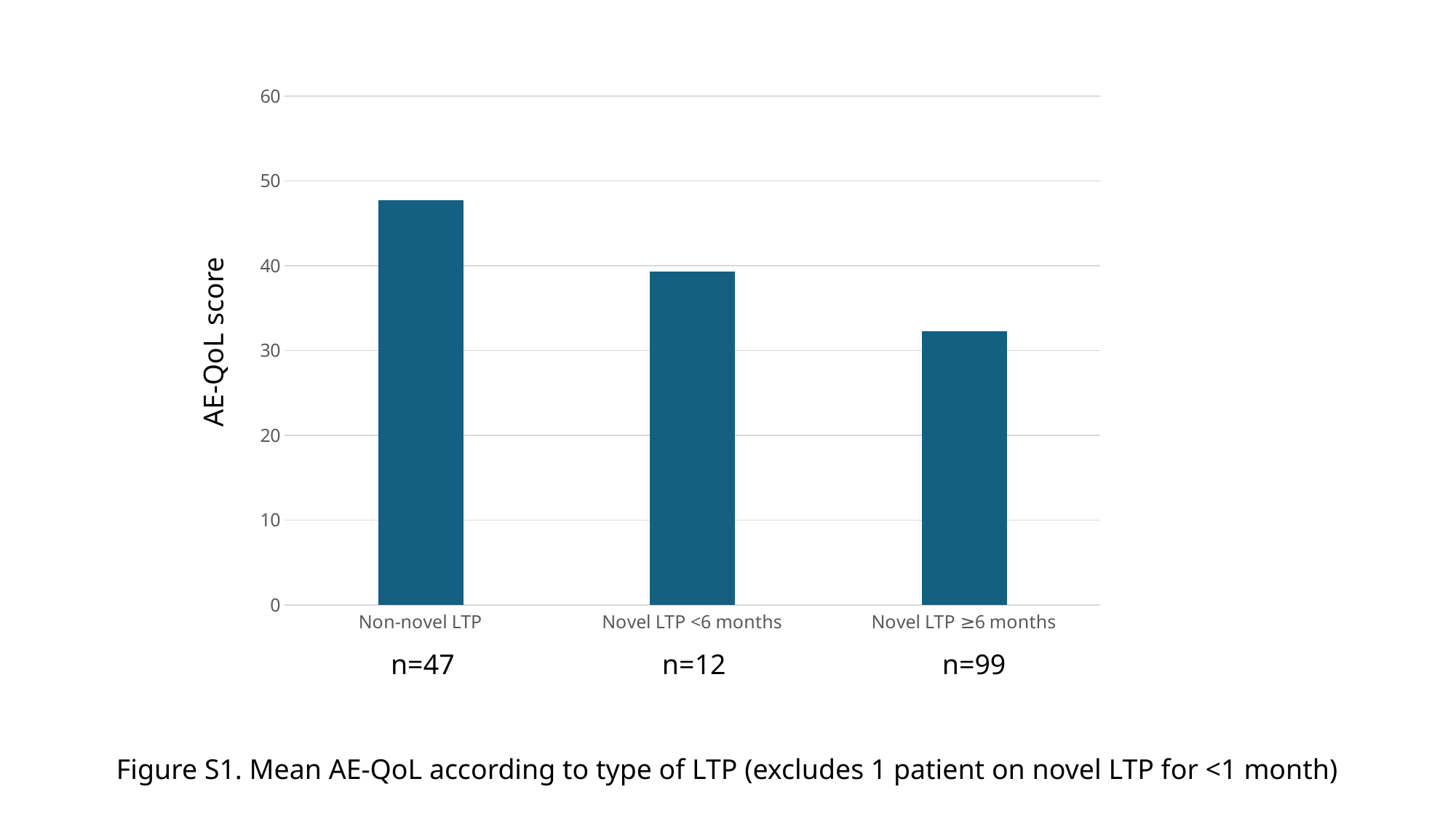

### Chart
| Category | AE-QoL score |
|---|---|
| Non-novel LTP | 47.7 |
| Novel LTP <6 months | 39.3 |
| Novel LTP ≥6 months | 32.3 |AE-QoL score
n=47
n=12
n=99
Figure S1. Mean AE-QoL according to type of LTP (excludes 1 patient on novel LTP for <1 month)
